# Language anxiety: understanding past research and new directions with d/Deaf, DeafBlind, and hard of hearing communities

**DOI:** 10.3389/fpsyg.2025.1558714

**Published:** 2025-04-10

**Authors:** Christina Kim, Laurel Aichler, Tiffany Bridgett, Onudeah Nicolarakis, Shilpa Hanumantha Lacy, Rachel Sortino, Poorna Kushalnagar, Rachel Pizzie

**Affiliations:** ^1^Educational Neuroscience Program, Visual Language and Visual Learning Research Center, Gallaudet University, Washington, DC, United States; ^2^Department of Linguistics, Gallaudet University, Washington, DC, United States; ^3^Department of Psychology, Gallaudet University, Washington, DC, United States; ^4^Department of Education, Gallaudet University, Washington, DC, United States; ^5^Center for Deaf Health Equity, Gallaudet University, Washington, DC, United States

**Keywords:** language anxiety, deaf, language proficiency, language deprivation, sign language

## Abstract

Language anxiety occurs when people associate negative emotional responses with using, expressing, or understanding language. In this review, we summarize past language anxiety research regarding specific language processes or subtypes: reading, writing, speaking, listening, and foreign language anxieties. Language anxiety is associated with poorer language proficiency and hinders learning and improving language skills. By conceptualizing language anxiety processes together, we identify common patterns and themes which will be vital for understanding how anxiety is detrimental to language performance. We discuss existing knowledge and propose applying theoretical framework names from another educational anxiety domain to more broadly understand language anxiety. These frameworks explain similar affective, cognitive, and behavioral relationships seen across subtypes of language anxiety. Past research suggests that some people are more likely to experience language anxiety and its detrimental effects on language. Through this review, we underscore the need for future directions to focus on individuals from diverse language backgrounds who are at greater risk for developing language anxiety. Social and linguistic factors, particularly in early life, foster negative emotional associations with and challenges to language acquisition. Future research collaborations with those who have lived experiences with language deprivation and language anxiety will clarify how emotion influences language development. We discuss how some d/Deaf, DeafBlind, and hard of hearing people have greater risk for developing language anxiety. Language anxiety is a prevalent, genuine barrier to learning and improving proficiency for deaf individuals who have difficulty acquiring language skills and experience adverse childhood communication experiences. Characterizing language anxieties toward signed and spoken languages will also clarify efforts to reduce anxiety for diverse language learners. Engaging underrepresented groups in language anxiety research can clarify how emotion plays a role in language development and identify groups that would benefit from future language anxiety-focused interventions. By focusing on and gaining a better understanding of emotional, diverse language experiences, we can build effective language anxiety interventions and improve language outcomes for all.

## Introduction

In addition to being places to develop cognitive skills and knowledge, educational environments are rich in social and emotional experiences that also inform how students learn. Repeated negative experiences within an educational context, or associated with a specific domain of learning, can lead to fear, anxiety, and avoidance (Pizzie and Kraemer, 2019). Academic anxiety refers to negative emotional responses like tension or anxiety associated with educational domains like mathematics, science, or language. Some students experience academic anxiety through fear, avoidance, or apprehension in specific situations, like taking tests or when using different academic skills. It is often associated with poorer performance, but even before formal tests are given, in some domains like math and reading, increased academic anxiety has been reported in children as young as first grade (Ramirez et al., 2019; Young et al., 2012). Regardless of age, academic anxiety is often related to physiological and emotional experiences. For example, a student can experience stress responses, like increased heart rate, sweating, and fast breathing (Jiménez-Mijangos et al., 2022; Pizzie and Kraemer, 2019), with anxious or uncertain feelings when doing arithmetic or trying to write an essay. A student might go “blank”—think more slowly, feel easily distracted, or experience disruption in executive function (Jalongo and Hirsh, 2010; Pizzie et al., 2020b).

But different kinds of academic anxiety not only have a negative influence because they *feel* negative, they also are associated with maladaptive strategies for learning, like avoidance or inefficient study strategies (Jenifer et al., 2022). Academic anxiety has a chicken-and-egg causal relationship with achievement in a particular domain; increased academic anxiety is associated with performance outcomes, e.g., decreased reading fluency or task performance, and decreased performance heightens academic anxiety (Ma, 1999; Pizzie and Kraemer, 2019; Ramirez et al., 2019). Likely, a bidirectional relationship exists that leads to a vicious cycle: weaker skills are related to more anxious feelings, and then more anxious individuals experience decrements in learning and poorer performance (Ramirez et al., 2018). Effects of such a cycle are seen in academic anxiety’s long-term detrimental relationship with educational and career choices (Daker et al., 2021). For example, a longitudinal study of first-semester university students found that over 4 years, math anxiety predicted lower STEM grades and students taking fewer STEM courses (Daker et al., 2021). This pattern held constant even after controlling for individual math ability, suggesting that academic anxiety contributed to long-term learning outcomes. Addressing academic anxiety in education is a priority, not only because it is associated with negative experiences for students, but because it relates to long-ranging consequences such as academic success and career choice. By conceptualizing academic anxieties together, like math anxiety or language anxiety, we can recognize patterns in emotional experiences and cognition, which will eventually lead to improved intervention strategies.

Some individuals develop a great deal of anxiety associated with language, or even specific language skills. Language Anxiety (LA) is a negative emotional response associated with using, learning, or understanding language (Argaman and Abu-Rabia, 2002; Daly et al., 1988; Horwitz, 2001; Pizzie and Kraemer, 2019). Past LA research has been specific to spoken languages and studied populations that had a foundational first language (L1) who were learning another language or to implement another language skill, such as learning the written form of the language (Dewaele et al., 2008; Horwitz, 2001; Ramirez et al., 2019; Szyszka, 2017). Two recent LA meta-analyses reflect the negative impact of LA on language performance. Teimouri et al. (2019) aggregated 216 correlations across 97 foreign and second LA studies and found a moderate, negative effect (*r* = −0.36) between LA and performance, measured by outcomes like course or test grade. Similarly, Zhang (2019) examined 46 foreign and second LA studies and further specified LA subtypes like reading anxiety and writing anxiety, finding an effect of *r* = −0.34.

In this review, we will focus on one domain of academic anxiety: LA. We discuss current understandings and propose theoretical framings from the math anxiety field that reflect relationships described in foreign LA studies. We also explore LA through its multiple subtypes—reading anxiety, writing anxiety, speaking anxiety, listening anxiety, and foreign LA—to highlight current knowledge. Doing so prefaces possible insights into LA and language development so that we can recognize harmful patterns and risk factors and compare across broader academic anxiety domains. By reviewing the extant literature on different LA subtypes, we seek to characterize LA in people at increased risk for developing it, like those who struggle to develop proficiency, as weak language skills can set the stage for and be a result of increased anxiety. In this review, we will explore past LA literature, mostly conducted in typically developing samples of participants. Further, we argue that future directions in LA research should prioritize studying samples that have more varied language backgrounds, as these individuals stand to benefit from work identifying and addressing factors that detract from language development.

We propose future directions in research with an underrepresented group whose members can have great risk and prevalence of LA. We assert that LA is particularly relevant to deaf people, and new research directions are needed where insights will come from collaborating with those who have LA-related lived experiences. For some deaf individuals, we predict that negative experiences with language and communication during childhood language development, or adverse childhood communication experiences, are related to increased language anxiety (Kushalnagar et al., 2020). By centering future studies within this subgroup of deaf and hard of hearing individuals, new LA insights will more broadly reflect bilingual and bicultural perspectives. Community-partnered research will create progress toward inclusive LA interventions and new knowledge that supports bilingual language acquisition for deaf communities and more.

## An overlooked, understudied population

Language supports cognitive development for higher-level features of learning, like social–emotional, incidental, and conceptual learning (Mayberry and Kluender, 2018; Werker and Hensch, 2015). People who experience additional challenges for acquiring language are more likely to develop LA due to decreased skill; the opposite is also true: people with more LA are less likely to develop proficiency due to anxious avoidance (Booth-Butterfield et al., 1991; Horwitz, 2017; Lee et al., 2020; MacIntyre, 2017). New LA directions should include deaf communities who have members at greater risk of developing LA associated with first or second language (L2) skills and who often encounter language inaccessibility. In 2023, over 12 million Americans were deaf or had a hearing disability (Thomas et al., 2024). Most deaf and hard of hearing babies in the U.S. are born to hearing parents, with estimates of 70–95% being born to at least one hearing parent (Mitchell and Karchmer, 2004). Unfortunately, many U.S. doctors and educational organizations still perpetuate debunked but common myths, like the idea that learning American Sign Language (ASL) will be a barrier to speech, or that bilingualism will confuse children and detract from language learning, when evidence suggests that bilingualism confers a host of benefits (Jasinska and Petitto, 2013; Kovelman et al., 2009; Nematova et al., 2024; Petitto, 2009; Petitto et al., 2012; Pontecorvo et al., 2023). As a result, many deaf and hard of hearing American children are born into families who might not have the ASL skills to fully expose them to a visually accessible language; these families require further training and support. Even the use of assistive devices like hearing aids and cochlear implants cannot guarantee full access to spoken language (Humphries et al., 2012). For U.S. families with deaf and hard of hearing children, often only a small percentage of family members learn ASL, with 72% of family members reporting that they do not sign ASL (Office of Research Support and International Affairs, 2014). Lack of early exposure to an accessible language puts these children at risk for decreased language proficiency and associated LA.

Chronic lack of access to natural language during the first few years of life is known as language deprivation (Hall, 2017). While it is uncommon in the mainstream U.S., it remains a prevalent concern among those who identify as culturally d/Deaf, DeafBlind, or hard of hearing (DDBHH) in addition to other intersectional and/or multicultural identities. DDBHH identity is intertwined with signed language and/or spoken language proficiency (Aldalur et al., 2021). DDBHH Americans often use ASL or English as their primary mode of communication and many have personal or proximal familiarity with language deprivation (Hall, 2017; Lane, 1989). Language deprivation is associated with a reduced ability to discriminate phonemes and communicate (Mayberry and Lock, 2003; Petitto et al., 2016). It can delay other cognitive skills, leading to long-term, negative linguistic and cognitive effects (Mayberry and Lock, 2003; Werker and Hensch, 2015). This means that many language-deprived DDBHH children enter school without sufficient school readiness skills and can struggle to express themselves and comprehend others. In fact, formal education (ages 4–5) is often the first time that some DDBHH American children gain exposure to ASL, at ages that are past the sensitive periods of language acquisition (Hall, 2017; Humphries et al., 2012; Mayberry and Kluender, 2018). Additionally, individuals who are constantly deprived of language access in their formative years run an increased risk for adverse childhood communication experiences and relatedly, developing LA.

According to Kushalnagar et al. (2020), adverse childhood communication experiences, specifically the direct child-caregiver communication construct (language deprivation), are psychosocial experiences during childhood, primarily with caregivers or family members, that result in constant and severe communication breakdown and exclusion from language and communication. From a cross-sectional national survey of over 1,500 DDBHH U.S. adults, researchers found that both direct (language deprivation at a 10% rate) and indirect adverse childhood communication experiences (communication neglect at a 30% rate) detract from interpersonal communication. Both are also positively related to an increased risk of multiple chronic health conditions. Consequently, adverse childhood communication experiences are adverse childhood experiences in this population (Kushalnagar et al., 2020). Among DDBHH people, such negative communication experiences increase LA vulnerability and heighten the risks of disrupted language learning. DDBHH people already face barriers to acquiring language given that many policies and practices discourage learning ASL in educational environments and communities (Nomeland and Nomeland, 2011). The additional complexity and prevalence of adverse childhood communication experiences that some DDBHH people experience compound the harms of reduced language access and proficiency, and increased LA (Hall, 2017; Hall et al., 2023; Humphries et al., 2012; Humphries et al., 2014). We will review the extant research on LA, which has mostly focused on typically developing populations of hearing people, and then expand on why future LA research is relevant to DDBHH communities.

## Language anxiety: current understanding

LA has two general perspectives about how emotional patterns manifest (MacIntyre, 2017; Szyszka, 2017). The first likens it to trait anxiety, a stable characteristic like general anxiety (Dewaele et al., 2008; MacIntyre and Gardner, 1991a; Szyszka, 2017). This suggests that LA appears in most contexts, whether they are language-related. The second perspective likens it to situation-specific anxiety (like a phobia, or test anxiety), happening only in certain contexts like in a classroom or when using the language (Horwitz, 2017; Horwitz et al., 1986; MacIntyre, 2017; Szyszka, 2017). This second perspective has become the predominant view (MacIntyre, 2017; Szyszka, 2017). Accordingly, in the short-term, language-anxious people experience temporary physiological changes like increased heart rate, muscle tensing, or sweating, and emotions like stress or uncertainty in particular contexts (Jiménez-Mijangos et al., 2022). Negative thinking about bodily and emotional responses leads to cognitive interpretations like “being overloaded” or “never getting it right” (Pizzie and Kraemer, 2019). People then learn and reinforce negative associations with the language context, becoming uncertain or afraid when interacting with others in a language. This aligns with our knowledge of anxiety and fear conditioning, in which reinforcing negative associations leads to increased vigilance and sensitivity to stimuli (Gazzaniga et al., 2013). Similarly, in the long-term, LA can lead to rigid beliefs about language skills and communication avoidance (Argaman and Abu-Rabia, 2002; Horwitz, 2001; MacIntyre, 2017; Szyszka, 2017). Socially, language-anxious people can use statements like “I just can’t learn other languages,” or “I’m bad at writing,” further reinforcing attitudes related to LA (Argaman and Abu-Rabia, 2002). Without intervention, language-anxious people learn to use fewer metacognitive strategies (Chow et al., 2017), or language-learning methods, and have poorer grades or task performance (Szyszka, 2017). They can also entirely avoid contexts where they will need to communicate in a specific language with others (Szyszka, 2017). Both short-term and long-term effects apply to a learner’s LA, regardless of domain scope.

### Theoretical accounts

Like how conceptualizing across LA subtypes and demographics will allow a broader understanding of LA, conceptualizing across educational domain theoretical frameworks will allow a broader understanding of academic anxiety. For example, reading anxiety researchers do not necessarily consider the broader picture of LA; focusing only on reading anxiety in a child’s L1 (Ramirez et al., 2019) misses similarities when considering reading anxiety in an adult’s L2. Considering overall LA patterns could lead to better interventions for more populations. Likewise, considering emotional experiences shared across academic anxiety domains, like LA and math anxiety, is vital to reveal broader patterns how anxiety disrupts academic performance. Math anxiety is a learned, negative emotional response to numerical content. Math anxiety and LA have their differences; for example, they have different social considerations and are subserved by different neural networks: frontotemporal-amygdala networks for language (Friederici, 2011; Jeong et al., 2016) and frontoparietal-amygdala networks for math (Young et al., 2012; Zhang, 2019). However, LA and math anxiety are both notable barriers to learning and context-driven, negative emotional experiences (Beilock and Maloney, 2015; Pizzie and Kraemer, 2019; Ramirez et al., 2018). Our math anxiety understanding comes from theoretical accounts that propose cause-and-effect relationships with math grades or task performance (Beilock and Maloney, 2015; Ramirez et al., 2018). Similar patterns exist in the LA literature but are specific to foreign language contexts (MacIntyre, 2017; Szyszka, 2017). We propose that foreign LA models can be both applied to broader LA contexts and simplified to use the same names from the math anxiety field: the Disruption Account—LA as a cause of worse proficiency, the Reduced Competency Account—LA as an effect, and the Bidirectional Account and Interpretation Account—LA as both cause and effect (Ramirez et al., 2018). Borrowing a theoretical name from another academic anxiety domain allows researchers to compare learning experiences and mechanistic explanations.

#### The Disruption Account

The Disruption Account framework, first named in the math anxiety literature, posits that negative emotions *cause* worse performance (Ramirez et al., 2018). Here, we use “Disruption Account” to reflect the idea from foreign LA models where LA causes worse proficiency (MacIntyre and Gardner, 1991b; Szyszka, 2017). Seminal foreign LA work showed that high LA students did worse on speaking and listening tasks while low LA students did better (Horwitz, 2001; Horwitz et al., 1986). This effect is hypothesized to occur by reducing working memory (Eysenck and Derakshan, 2011). Working memory involves mentally holding a small amount of easily accessible information; its capacity plays an important role in recalling encoded information and processing semantics (Shin, 2020). In a meta-analysis across 25 studies, Shin (2020) found a medium effect size (*r* = 0.30) between L2 reading comprehension and working memory. This suggests that lower LA relates to better language performance because of increased working memory (Ramirez et al., 2019; Taboada Barber et al., 2021). In this manner, working memory and LA trade off; when a person has low or no LA, they have more working memory resources available for executive functions and language processing. Conversely, when a person experiences high LA, working memory becomes overwhelmed and language processing decreases (Chow et al., 2021; Moran, 2016). Recent reading anxiety work found a similar negative impact on language performance (Chow et al., 2021; Shin, 2020). Yet this overall impact depends on the extent of LA. For example, one study’s slight positive relationship between reading anxiety and motivation suggests that some language-anxious students use low levels of LA to engage with content (Zarei, 2014). Beyond that non-specific threshold however, in line with the Disruption Account, disrupted executive functions like poorer inhibition lead to reduced engagement and increased task-switching (Pizzie and Kraemer, 2019; Teimouri et al., 2019). The well-studied, negative relationship between LA and performance is driven by students with high-LA who show high motivation to avoid content like halting interactions in a group conversation (Horwitz et al., 1986).

#### The Reduced Competency Account

The Reduced Competency Account framework from the math anxiety field suggests that academic anxiety is an *effect* of poor performance (Ramirez et al., 2018). Similarly, foreign LA models argue that LA comes from weak language skills (Argaman and Abu-Rabia, 2002; Ganschow and Sparks, 1996; Sparks, 1995; Tran, 2012). For example, Ganschow and Sparks hypothesized that if worse task performance occurred in non-communication skills like reading, poor scores were independent of emotion and caused by worse language skill (Ganschow and Sparks, 1996). They studied 154 female high schoolers grouped by low and high LA and only found differences in reading comprehension, asserting that LA is an effect of poor language learning. Ganschow and Sparks saw further support for this argument as skill deficits like dyslexia in a native language associated with poorer proficiency (Ganschow and Sparks, 1996), independent of emotion (Szyszka, 2017). Absent of disabled reading, a recent adolescent reading anxiety study also found evidence for LA as an effect of poorer skill. Ramirez et al. (2019) showed that decreased achievement in autumn predicted increased reading anxiety levels in the spring. Weaker skills are correlated with increased LA, however, it seems overly simplistic to assume that individuals with LA are just less skilled to begin with. Furthermore, as reviewed by MacIntyre (2017), LA has impacts beyond aptitude that are worth studying.

#### The Bidirectional and Interpretation Accounts

As proposed by math anxiety researchers (Ramirez et al., 2018), the Bidirectional Account framework suggests that LA is *both a cause and effect* of decreased performance. This relationship is similarly described in reviews of the foreign LA literature (MacIntyre, 2017; Szyszka, 2017; Tran, 2012). Recently, Ramirez et al. (2019) showed evidence for this bidirectionality by studying reading anxiety. In addition to finding that decreased reading performance predicted more reading anxiety in the spring, their work with monolingual first and second grade readers also found that fall reading anxiety predicted worse spring reading performance. Extending the Bidirectional Account, the Interpretation Account framework additionally considers individual interpretations of LA contexts (Ramirez et al., 2018). The foreign LA literature outlines a similar concept, where LA dynamically depends on the situation, whether a person learns to associate LA with motivation, and whether they alter beliefs toward language efficacy, negative self-talk, or communication (MacIntyre, 2017; Wang et al., 2024). The Interpretation Account framework considers cause and effect relationships between LA and performance and additional variables from dynamic systems theory like complex individual differences in reappraisals, or interpretations (Ramirez et al., 2018; Wang et al., 2024).

## Language anxiety subtypes

### Reading anxiety

Reading anxiety involves visually processing text information (Chow et al., 2021; Jalongo and Hirsh, 2010; Ramirez et al., 2019; Taboada Barber et al., 2021). It can include physical responses like tension and emotions like embarrassment, fear, or confusion (Jalongo and Hirsh, 2010). It is also tied to beliefs like thinking one is, “bad at reading” (Ramirez et al., 2019) and can lead to avoidant behaviors (Jalongo and Hirsh, 2010). Work with young adult L2 learners has linked higher reading anxiety levels with lower reading grades and test scores. A reliability study by Campbell and Shaw (1994) with military foreign language learners, many of whom just graduated high school, correlated a self-reported reading anxiety measure with a required language placement test. Similarly, a mediation study by Chow et al. (2021) modeled reading anxiety as a mediator between verbal working memory and English reading comprehension. Both studies’ negative associations between reading anxiety and test scores reflect a Disruption Account framework. Similarly, a one-year longitudinal study with monolingual children (Ramirez et al., 2019) and a cross-sectional study with emerging bilingual children (Taboada Barber et al., 2021) both examined reading acquisition and also showed a negative relationship. Ramirez et al. (2019)‘s study additionally found evidence for reading anxiety as an effect of weaker performance. Given this, and a gender effect where young boys and men had more anxiety and negative performance impacts from stereotype threat (Ramirez et al., 2019; Campbell and Shaw, 1994, respectively), the Interpretation Account framework better describes reading anxiety’s variance in individual differences and self-interpretation. Ramirez et al. (2019) recommended addressing reading anxiety with positive self-talk, cognitive reframing, and deep breathing, as well as adapting instruction style to better engage learners (Ramirez et al., 2019). These interventions could benefit language-anxious readers across ages and populations.

### Writing anxiety

Writing anxiety refers to encoding a message into a written form (Cheng, 2004). Studies with adolescents and adults support a Disruption Account framework, where increased writing anxiety related to decreased writing performance. In working with 70 Israeli adolescents (12–13 years old), Argaman and Abu-Rabia (2002) compared self-reported scores from an adapted version of the Foreign Language Classroom Anxiety Scale with scores on a brief written English task. Greater writing anxiety related to worse scores, and some students felt so much anxiety that they erased all their written work. Studies with older samples also reflect this negative relationship. An early study by Cheng et al. (1999) gave 433 Taiwanese university English majors a Chinese-translated version of the Second Language Writing Apprehension Test and found that English writing components of self-confidence and evaluation apprehension were negatively related to written course grades (*r* = −0.25 and *r* = −0.13, respectively). Huerta et al. (2017) studied 174 U.S. graduate research students, 96 of whom were non-native English speakers, enrolled in a supportive academic program teaching writing strategies and resources. Self-reported measures showed that writing anxiety was most predicted by a female gender, a non-native English background, decreased self-efficacy, and no prior writing program exposure. Qualitative research supports the negative association observed in quantitative studies. For example, Cheng (2004) surveyed 67 foreign language university students majoring in English and additionally conducted 27 semi-structured interviews to learn about people’s writing experiences writing. Many of these native Chinese speakers shared decreased writing performance and increased physiological and emotional responses to increased writing anxiety, like headache, sweating, frustration, uncertainty, fear, and low self-confidence. Other writing-anxious people experience cognitive aspects including beliefs like, “I make many spelling mistakes” (Argaman and Abu-Rabia, 2002). Long-term writing anxiety risks reduced motivation, poorer self-efficacy or self-confidence in writing ability, and an unwillingness to write, as well as avoiding writing opportunities and advanced writing courses (Argaman and Abu-Rabia, 2002; Huerta et al., 2017). Proposed writing anxiety interventions focus on affect like encouraging self-regulation (Huerta et al., 2017). Reframing anxiety through expressive, non-graded work can scaffold language development while reducing fear about writing.

### Speaking anxiety

Speaking anxiety, separate from that attributed to social anxiety, occurs in contexts like speaking in a particular language (Cheng et al., 1999; Erdiana et al., 2020; Horwitz et al., 1986). Past research, excluding public speaking anxiety which is considered a subtype of trait or social anxiety (Blöte et al., 2009), predominantly examines foreign language learners. Such work shows a negative relationship in line with the Disruption Account framework, where increased speaking anxiety predicts decreased speaking performance (Cheng et al., 1999; Horwitz et al., 1986; Leong and Ahmadi, 2017). For example, an early study by Horwitz et al. (1986) that looked at the reliability of the Foreign Language Classroom Anxiety Scale with 75 university students taking introductory Spanish classes showed contextual instances of speaking anxiety specific to foreign language use. Another study of 433 Taiwanese English majors by Cheng et al. (1999), first mentioned in the writing anxiety subsection, also gave students the Foreign Language Classroom Anxiety Scale. Cheng et al. (1999) found that the English speaking self-confidence component negatively related to speaking course grade (*r* = −0.19). Other work suggests that speaking anxiety occurs in adolescence. Though a study of eighth grade Indonesian English language learners by Erdiana et al. (2020) that used an adapted speaking anxiety measure did not examine speaking proficiency, 18 of 29 students reported having at least moderate speaking anxiety. Short-term arousal from speaking anxiety can present as increased heart rate or blood pressure and feelings of timidness, fear, or confusion (Erdiana et al., 2020; Leong and Ahmadi, 2017). People with speaking anxiety experience forgetfulness, decreased motivation, and negative self-perception, and avoid verbal language use (Erdiana et al., 2020; Leong and Ahmadi, 2017). Proposed interventions seek to improve student attitudes and learning environments by adjusting content to match student interests, offering penalty-free opportunities, and encouraging language expression (Leong and Ahmadi, 2017).

### Listening anxiety

Listening anxiety, or listening comprehension anxiety, occurs when spoken words are perceived as too difficult or unfamiliar, yielding low self-confidence in understanding (Atasheneh and Izadi, 2012; Dalman, 2016; Kim, 2000; Zhang, 2013). Like speaking anxiety, listening anxiety has often been studied in foreign or L2 classrooms and negatively relates to language comprehension, following a Disruption Account framework (Vogely, 1998; Zhang, 2013, 2019). A meta-analysis by Zhang (2019) aggregated correlation effects across 46 studies that measured LA and performance, and found that listening anxiety compared to other LA subtypes had the greatest impact on associated skill performance. The same study also found that listening anxiety did not improve with listening proficiency; anxiety levels were stable across beginning, intermediate, and advanced proficiency levels (Zhang, 2019). As such, reducing listening anxiety is likely to improve other areas of language comprehension. A study of 110 Iranian university students taking listening tests from the standardized Test of English as a Foreign Language showed that listening-anxious students experienced muscle tension and felt afraid, nervous, or uneasy (Dalman, 2016). Relatedly, from a qualitative study with 140 American university students after a listening portion of a Spanish language exam, students can learn to feel less confident in language-listening contexts and worry about judgment from others (Vogely, 1998). More research is needed to determine whether gender factors into listening anxiety (Atasheneh and Izadi, 2012). Interventions found to reduce listening anxiety and increase performance focus on creating more supportive environments (Kim, 2000; Vogely, 1998; Zhang, 2013). For example, suggestions included adding visuals, providing positive feedback, encouraging reflection (Kim, 2000), allowing multiple playbacks, and teaching students to strategically process spoken content (Vogely, 1998). These interventions can provide students with scaffolding to reduce LA and increase accessibility in multiple learning contexts.

### Foreign language anxiety

As shown in earlier subtypes, foreign LA can include reading, writing, speaking, and listening anxiety in a non-native language. Sometimes LA and foreign LA are distinguished, and other times they are combined. These variable uses depend on context, like number of languages, formal classroom language learning, and formal or informal L2 learning where the language is required to function in society (Argaman and Abu-Rabia, 2002; Dewaele et al., 2008; Horwitz, 2001; Szyszka, 2017; for additional nuance and a discussion on multilingualism complexity, see Dewaele et al., 2008; Ganschow and Sparks, 1996; Horwitz, 2001; Horwitz et al., 1986; Sparks, 1995). As reviewed by MacIntyre (2017), past work shows evidence for foreign LA as both a cause (Horwitz et al., 1986; e.g., Disruption Account) and an effect (Ganschow and Sparks, 1996; e.g., Reduced Competency Account). Meta-analyses that included both “foreign language anxiety” and “second language anxiety” in their systemic literature reviews found overall moderate, negative relationships between foreign LA and proficiency (Teimouri et al., 2019; Zhang, 2019). This supports the idea of foreign LA fitting a Bidirectional or Interpretation Account framework. A large part of the foreign LA literature comes from foreign language classrooms with older adolescents and adults (Atasheneh and Izadi, 2012; Chow et al., 2021; Dalman, 2016; Erdiana et al., 2020; Horwitz et al., 1986; Kim, 2000; Kim and Kim, 2004; Leong and Ahmadi, 2017; MacIntyre, 2017; MacIntyre and Gardner, 1991a; Teimouri et al., 2019). That said, studies with younger second language learners such as children and adolescents suggest similar foreign LA phenotypes and impacts on language performance (Taboada Barber et al., 2021; Tuncel et al., 2020; Xu, 2023). Regardless of age or language, the foreign LA literature is dominated by research on people with early exposure to an accessible L1.

Studies show addressable patterns of increased physiological and emotional arousal related to foreign language skills like dry throat, frustration, and fear (Argaman and Abu-Rabia, 2002; Horwitz, 2001; MacIntyre, 2017; Onwuegbuzie et al., 1999; Szyszka, 2017; Toyama and Yamazaki, 2021). Learned contextual associations with negative responses lead to going “blank,” low self-efficacy, and reluctance to interact with proficient speakers (MacIntyre, 2017; Onwuegbuzie et al., 1999; Szyszka, 2017; Toyama and Yamazaki, 2021). In the long-term, maladaptive behaviors can develop like inattention, sleep disturbances, procrastination, over-studying, understudying, skipping class, and negative self-talk (Horwitz, 2017; Huerta et al., 2017; Onwuegbuzie et al., 1999). Effective interventions address affective and contextual factors. For example, recognizing foreign LA as a legitimate experience, providing sensitive feedback, and offering sincere teacher support best mitigated negative outcomes (Horwitz, 2017). Other supportive interventions include journaling, testing more socially demanding and perhaps more anxiety-provoking skills like speaking or listening separately, and promoting enrollment in more language classes (Horwitz, 2001; Onwuegbuzie et al., 1999; Toyama and Yamazaki, 2021). Interventions meant to increase motivation through gamification showed some success with mixed results; for example, Toyama and Yamazaki (2021) found inconsistent evidence for computer-mediated game interventions. No matter the intervention, the evidence supports reducing foreign LA to ameliorate negative impacts on language learning and performance.

## Language anxiety risk factors

From reviewing studies of LA subtypes with typically developing populations, unsupportive learning environments, initially weaker language skills, and inflexible, negative self-beliefs increase the risk of developing LA. Factors that detract from early language development also likely contribute to developing a vicious cycle of LA because they predispose risk factors and amplify their impacts. While heightened LA can develop at any age and at any language skill level, older language learners have more emotion regulation strategies to protect against negative outcomes. During infancy, years known as the sensitive language acquisition periods, our brains are ready to acquire any language (Petitto, 2009). Later age of exposure to a language heightens the risk of LA, as learning a language outside of sensitive language acquisition periods changes the mechanism by which individuals learn language. Instead of picking up statistical regularities in natural language input as infants do (Petitto, 2009), people with later age of exposure may need to acquire language through formal instruction. Additionally, mechanisms that support language differ from those utilized from early age of exposure, as shown by neuroimaging studies (e.g., Nichols et al., 2021).

For early childhood L1 acquisition, insufficient early language input is associated with significant negative consequences for language and cognitive outcomes (Petitto et al., 2016; Taboada Barber et al., 2021; Werker and Hensch, 2015). Poor input from inaccessible language environments make it difficult to develop proficient language skills. For example, sparse (Romeo et al., 2018) and inaccessible (Hall et al., 2023; Kushalnagar et al., 2020) language input in a child’s home environment can hinder typical language growth and diminish motivation and engagement (Horwitz, 2017; MacIntyre, 2017; Ramirez et al., 2019; Szyszka, 2017; Zarei, 2014). For a sample of DDBHH Canadians, lack of early access to a visual signed language was associated with decreased gray matter volume in frontotemporal brain regions in adulthood. By contrast, DDBHH adults who had been exposed to ASL during infancy did not show the same decrease in frontotemporal gray matter volume (Cheng et al., 2023). Such impoverished language environments promote LA and negatively impact cognitive and behavioral development (Nematova et al., 2024; Romeo et al., 2018).

But improving early life language conditions can mitigate long-term risks (Hall, 2017; Nematova et al., 2024; Romeo et al., 2018). Enriching language environments positively impact both behavior and brain structure. Children who had more conversational interactions with a caregiver, regardless of socioeconomic status, showed stronger white matter tract connectivity in brain areas supporting expressive and receptive language skills (Romeo et al., 2018). As previously mentioned, DDBHH children who access language through assistive devices like hearing aids or cochlear implants may not have enough sound fidelity to acquire spoken language using assistive devices without additional language support (Humphries et al., 2014). However, DDBHH children with cochlear implants who had earlier age of exposure to a visual signed language also had strong spoken language processing, offsetting potential harms from language deprivation (Nematova et al., 2024). Early age of exposure and plentiful, accessible language input can protect against conditions that set the stage for developing LA and weaker proficiency. Addressing LA has the potential to ameliorate negative effects of early childhood language adversity. It also addresses important behavioral motivations both in childhood and adulthood, as LA can develop throughout the lifespan.

## Why is language anxiety relevant to DDBHH people?

Sociocultural factors that contribute to and perpetuate language deprivation and reduced proficiency in DDBHH people heighten risks for LA and lowered language learning aspirations (Hall, 2017; Hall et al., 2023; Humphries et al., 2012; Kushalnagar et al., 2017, 2020). Additionally, some DDBHH Americans have unique experiences of LA related to English language anxieties. U.S. DDBHH English learners draw parallels to English foreign language learners, who learn a language outside of the sensitive periods of language acquisition, but there are unique considerations given an impoverished L1 (Twomey et al., 2020) and qualitatively different reading approaches from both English monolinguals and English second language learners (Cooley et al., 2025). Additionally, many DDBHH Americans struggle with reading proficiency and related reading anxiety, lagging behind their hearing peers in developing language skills. The average U.S. DDBHH high school graduate reads and writes at about a fourth-grade level (Mayer, 2012; Mayer and Trezek, 2020; Traxler, 2000). DDBHH stereotypes and expectations related to poorer English proficiency can contribute to decreased self-efficacy, negative emotions, and lower aspirations. From the Interpretation Account framework, these language-anxious people experience intrinsic and extrinsic pressures that can detract from their language skills and emotional wellbeing. This appears to parallel the current state of LA research which focuses on studying hearing samples and spoken languages, but we cannot assume past LA findings apply to DDBHH populations without more research. For example, “speaking” anxiety, for some DDBHH individuals, can be akin to “presentation anxiety” in spoken or signed languages, but is uniquely different than foreign language speaking anxiety. Presentation anxiety in DDBHH people may similarly disrupt self-expression and result in negative feelings, but will vary on an individual’s language preference and whether they choose to speak English and sign ASL. Accordingly, expanding the scope of LA research to use broader language process-specific terminology, consider signed languages, and study those with impoverished L1s will lead to more holistic understandings.

For the purposes of this review, we additionally considered LA-*related* studies with DDBHH samples. Even so, only five relevant studies described LA-like experiences and none used LA terminology. That said, all five studies suggest a genuine prevalence of LA-related experiences in DDBHH communities and a need for more LA research with this population. Notably, these studies show that for DDBHH people, adverse childhood communication experiences can predispose LA, but are not required to develop LA. Previous related work in this population suggests a genuine prevalence of LA and need for more research. Searches through Education Resources Information Center, Language Learning Behaviors Abstracts, and ProQuest Dissertations and Theses databases, and Google Scholar with the keywords *deaf, dhh,* and *sign language*, and *language anxiety, apprehension, reading anxiety, writing anxiety, speaking anxiety, expressive anxiety, listening anxiety, receptive anxiety, foreign language anxiety*, and *second language anxiety*, returned few studies about signed languages. Initial LA-related results mostly referenced hearing L2 learners (Bajkó and Kontra, 2008; Dewaele et al., 2008; Domagała-Zyśk and Kontra, 2016; Shaw and Hughes, 2006; Webb et al., 2024). Other results returned only five publications with deaf samples that were relevant to language anxiety or similar experiences. Each examines the role of emotion in language contexts and replicates the same negative relationships seen in past work with LA and long-term outcomes.

The earliest study by Booth-Butterfield et al. (1991) explored how deaf students’ anxiety associated with communicating in ASL influenced their sign effectiveness or understandability. This anxiety encompassed two concepts: predispositional anxiety, or apprehension toward a communication mode (ASL), and trait communication anxiety, “the involvement of trait anxiety in…signing communication.” Predispositional anxiety is also described as “talk” anxiety, like a situation-specific anxiety occurring only when students express themselves in ASL. Booth-Butterfield et al. hypothesized that predispositional anxiety about communicating would negatively impact signing effectiveness—a well-replicated pattern across the LA literature. For example, they found significant negative correlations between higher predispositional anxiety and less clear signing (*r* = −0.28), smaller signs (*r* = −0.23), less intense signs (*r* = −0.22), slower signs (*r* = −0.37), and incomplete communication (*r* = −0.27). These correlations are similar to the moderate, negative trends seen in foreign language anxiety meta-analyses (*r* = −0.36, Teimouri et al., 2019; *r* = −0.34, Zhang, 2019). This work suggests that anxiety about signed communication in deaf participants was also related to communication skill in ASL (Booth-Butterfield et al., 1991).

By giving ASL-adapted measures of communication anxiety and communication fear to 42 DDBHH American adolescents in seventh through twelfth grade, Booth-Butterfield et al. (1991) studied a construct most like speaking anxiety. These “talk” anxiety scores were correlated with teacher-rated signing effectiveness on sign clarity, intensity, speed, size, the use of gestures and facial expressions, and sentence completeness. From the researchers’ definitions of predispositional and trait communication anxieties, however, it is difficult to tell whether trait or situation-specific communication anxieties were measured. This is further obscured by their suggestions of interventions, like systematic desensitization and biofeedback, which address trait and situational anxieties, respectively. Many of the items included in Booth-Butterfield et al. such as “In meetings I am afraid to talk,” and “When I communicate I feel afraid,” are difficult to distinguish from social anxiety. Notably, this study is the only LA-relevant study with a DDBHH sample of adolescents. Despite its limitations, the incomplete signing style seen in the communication-anxious DDBHH students studied suggests that LA and avoidant behaviors are evident and observable at an early age in this population.

Another U.S. study that supports a need for more LA research in this population explored how computer-mediated communication in a writing course supports DDBHH college students’ written English development. This study was based on L2 acquisition research, because written English was the L2 of the ASL signers in this study. Carlson (2004) showed that despite measured benefits from computer-based communication during the course, decreases in writing quality after the course were, in part, related to language anxiety. The DDBHH students did a pre-course writing sample at the beginning of the semester during a “low anxiety state,” and a post-course writing sample which was the state exit testing that “imposed a degree of apprehension on the students;” where two failed attempts on the written exit test meant retaking the class. Carlson’s qualitative analysis showed that students expressed “writer anxiety” from needing to pass the writing test and experienced negative score impacts seen in second language anxiety research (Horwitz et al., 1986). This study effectively showed both the subjective impacts of LA in a DDBHH sample from analyzing computer-based instructor and peer comments on written work, and the objective impacts on writing scores, measured by the Revised Test of Ability to Subordinate, a syntactic written English test for deaf college students, and the ESL Composition Profile, where three raters scored facets of writing. This study did not include a measure of emotional language experiences aside from qualitative work; if it had, this would be most comparable to writing anxiety. It seems that LA disrupts DDBHH students’ written English proficiency; more work is needed to add to our knowledge of writing anxiety in DDBHH populations.

For example, Lee et al. (2020) explored the feasibility of using psychophysiological methods to measure presentation anxiety in DDBHH people within a naturalistic language environment that had multiple sociocultural factors. The DDBHH students presented both individually and with a hearing teammate to a hearing audience in a non-native sign language, facilitated by ASL voice interpretation. Factors like multilingualism, multiculturalism, and being in a foreign language classroom context generated an anxiety-provoking scenario for the signers. While the DDBHH students presented in ASL, their second signed language, researchers measured electrodermal activity data; after each presentation, students completed semi-structured interviews and after all presentations, and self-reported LA via the Foreign Language Classroom Anxiety Scale. Specific situations for the language-anxious DDBHH signer, like fingerspelling, forgetting an ASL sign, and eye contact with a hearing instructor were associated with feelings of humiliation and anxiety, as well as with electrodermal spikes, or increased sweat and autonomic arousal levels (Lee et al., 2020). Though this study only had two participants and used a LA measure that has been critiqued on whether it measures foreign LA or general anxiety (Aida, 1994), this work shows that negative emotion is disruptive to language proficiency and performance.

Qualitative data from Aldalur et al. (2021) further support links between sociocultural, linguistic, and LA experiences in DDBHH people. DDBHH acculturative stress, or tension experienced during the process of adapting to two or more cultures, i.e., hearing and Deaf cultures, was found to be intertwined with language proficiency and feelings of belonging (Aldalur et al., 2021). People with self-reported weaker language proficiency in either ASL or English tended to experience greater acculturative stress and subsequent effects, like avoiding situations that used a certain language or thinking dissonant thoughts when considering their language communities and identities. From focus groups with 13 people, Aldalur et al. (2021) highlighted language experiences associated with acculturative stress, like having limited access, avoiding asking for clarifications, being expected to accommodate hearing people and conversation styles by lip reading or reducing expressiveness, being left out of conversations, and identifying social groups based on fluency or language choice. There are clear LA experiences and unique sociocultural considerations in DDBHH communities, and a need to measure these experiences in an accessible way.

Aldalur and Pick (2022) proceeded to create a measure of Deaf acculturative stress, the Multidimensional Inventory of Deaf Acculturative Stress, which includes items about negative emotion and communication. Items like, “I am frustrated that my family never learned sign language,” “I often “fake it,” or pretend that I understand conversations in spoken English when with hearing people,” and “It bothers me that I do not sign ASL like a native” evoke LA-like experiences. Such items appear to measure LA in DDBHH communities and were developed from DDBHH people. This online study with 104 DDBHH participants was administered in written English and validated with the Deaf Acculturation Scale. The use of this measure lends further evidence to LA being a prevalent research area for DDBHH communities but does has some limitations. For example, the measure is only available in written English so DDBHH signers who have reduced proficiency in English would have benefitted from accompanying ASL videos. The results of this study suggest that some DDBHH individuals experience acculturative stress, which is likely related to LA and additional sociocultural factors, reflecting genuine emotional experiences of bimodal bicultural DDBHH communities. While the existing DDBHH work discussed in this section is small and has some limitations, it also shows opportunities to gain new insights to LA in this population. By expanding LA research to include advanced neuroimaging technologies and statistical approaches, we can learn more about how bilingualism, language learning, and the neural correlates of language are influenced by emotion.

How then, might we mitigate potential language and communication risks? Factors like delayed age of ASL exposure can put DDBHH American children at risk for decreased language access and explain the achievement gap between DDBHH children and their hearing peers (Hall, 2017; Pontecorvo et al., 2023). However, providing access through ASL reduces risks of language deprivation, adverse childhood communication experiences, and potentially LA. For example, White (2019) analyzed a subset of American deaf and hard of hearing, lifelong hearing aid and cochlear implant users who only used spoken English. This group showed decreased language task accuracy, slower responses, and higher self-reported cognitive effort compared to typically hearing peers matched on intelligence and language comprehension. Complementing this finding, Nematova et al. (2024) found that American cochlear implant users who had early age of exposure to ASL showed greater activation in classic language processing areas of the brain; learning ASL did not impede, but rather, supported their bilingual language acquisition. For children acquiring a L1, supportive and accessible language environments scaffold typical, native-like socioemotional and cognitive processes. As Emmorey et al. (2013) and Twomey et al. (2020) found from fMRI studies with deaf and hearing groups matched on signed and written language proficiency, DDBHH signers with early age of sign exposure showed expected, native-like language processing in the superior temporal cortex, a language network observed in native hearing speakers. In contrast, individuals with later age of sign exposure showed more visuospatial processing in the parietal lobe (Twomey et al., 2020). Early sign language age of exposure confers benefits for native language processing for DDBHH people regardless of later-gained proficiency.

Because DDBHH identities and language backgrounds are incredibly diverse (Aldalur et al., 2021; Atkinson et al., 1998; Lane, 1989; Leigh, 2009; see “An overlooked, understudied population” section for additional detail), we propose that DDBHH people also vary in LA associated with each language and specific language processes. We predict that this is attributed to varied experiences and proficiencies across signed, spoken, and written languages. Importantly, adverse childhood communication experiences are a predisposition, not a prerequisite, for DDBHH people to develop LA. Systemic educational and cultural barriers detract from language development for all DDBHH people, creating problems for developing language skills and proficiency. Because of the intertwined relationship between LA and language skills, barriers that weaken language acquisition are also likely related to increased LA. ASL is a minority language in the U.S., so DDBHH Americans often experience societal expectations to develop proficiency across both ASL and English (Atkinson et al., 1998; Lane, 1989; Leigh, 2009) in addition to other languages in their communities. Accordingly, DDBHH people likely experience varying levels of increased anxiety associated with different skills and languages. Not all DDBHH people will develop increased LA, and individual differences observed in past research with hearing people, like age of exposure and learning environments (MacIntyre, 2017), will play a huge role in developing anxiety associated within or across language modalities. Addressing the systemic barriers to language acquisition for DDBHH people is a key priority. Additionally, considering emotional and social experiences that detract from language development will provide important support for language skills.

## Discussion

Over the past several decades, researchers have progressed in understanding LA’s causes, effects, and individual differences. Shared themes underlying past work suggest that LA looks and feels similarly across different language skills and demographics. A broader conceptualization will shed light on how negative emotion disrupts language development. There is a clear need for more LA studies with signed languages and people that experience early and prevalent barriers to language access. That said, experiencing early barriers to language development predispose but do not preclude developing LA. The variety of unique experiences, modalities, and sociocultural considerations that DDBHH people have offers opportunities to describe and address anxiety in diverse language learners.

Thoughtful, collaborative studies with DDBHH people will clarify whether well-studied LA interventions for hearing learners can also benefit DDBHH learners. Collaborating with DDBHH people with lived adverse childhood communication experiences will present opportunities to design interventions with language accessibility and potentially mitigate the impact of LA. Importantly, this provides an opportunity for DDBHH individuals to not only be included as research participants, but to be included as team members and provide meaningful feedback for research designs. In a similar, collaborative spirit, mainstream educational systems can partner with educator training programs that include and feature DDBHH teachers who may already implement accessible strategies. Moreover, partnerships like this represent an way to provide high-quality research training and foster trust between the research community and DDBHH communities. For example, evidence-based interventions like emotion-regulation training to reduce anxiety (Pizzie et al., 2020a) could be made available in both ASL and English and then evaluated with DDBHH American children to assess the training efficacy and effectiveness. Many young DDBHH and hearing children struggle with learning to read, often with pedagogy that focuses on sound-based phonology. By addressing anxiety about reading and building strategies that rely more on visual language and communication, we can tailor strategies according to the learner’s needs and ensure that the strategies are made to reduce anxiety and benefit a variety of language learners.

Interventions designed with creativity, neurodiversity, and language accessibility in mind will generate new educational and learning practices that address diverse needs. Accessible interventions for DDBHH learners that promote language development require systemic change for widespread implementation and should prioritize early language exposure. For example, ASL-English storybook applications offer DDBHH children early exposure to stories in simultaneous ASL and English (Herzig and Allen, 2023). These storybook apps have been adapted to several other signed languages. Hearing parents reading these stories with their DDBHH child can confer bilingual language development benefits for each other. Furthermore, by addressing one-on-one child and caregiver communication issues that negatively impact language skills, alternate interventions that provide access to ASL will address negative experiences that contribute to LA.

Even with available interventions that directly support early and accessible language, people can still develop LA at various ages. Their levels of language proficiency across modalities can also vary, particularly with a less accessible language modality, separate from psycho-cognitive factors. Targeting LA in language interventions will help address underlying key emotional factors that detract from language-learning motivation and proficiency. By tailoring strategies to reduce anxiety, we can create a supportive environment that fosters confidence and enhances language acquisition. For example, a vanishingly small percentage of hearing family members of DDBHH American children learn ASL as a L2 (Office of Research Support and International Affairs, 2014). Hearing family members can also experience LA associated with learning a L2 and avoid language learning opportunities. Thus, addressing avoidance and LA in hearing family members would encourage more families to learn ASL, providing a more accessible and supportive language environment for DDBHH children. Educational policy makers can support these families by offering training and support on educational advocacy, representing these families’ needs and priorities on local and regional scales, and providing early support to locate community resources. Additionally, though addressing early language interventions is a priority, we must also address the deleterious effects of LA in individuals who have already developed negative relationships with languages and specific skills. Many children struggle with learning to read and write; interventions that address both emotion and language context benefit a wide variety of learners by addressing a spectrum of individual differences in LA. Previous research on LA shows that it has a wide variety of significant negative impacts on language learning and performance. Therefore, addressing the negative influence of increased anxiety on language is an important priority.

### Where should language anxiety research go next?

LA research has and will continue to provide important insight into factors that bolster language learning; partnering with DDBHH people and training them on research skills to study LA will offer new insights into language development. The extant LA literature covers a variety of skills but presents a limited picture of bilingualism by emphasizing spoken language learners with established L1s. More research is needed to build on existing LA-related studies regarding how LA relates to proficiency in a signed modality and what LA looks like in those with an impoverished L1. Considering LA among DDBHH people with lived experiences will allow us to characterize or measure affective influences on long-term cognitive, linguistic, and socioemotional outcomes. New inroads to understanding human language will come from research partnerships that explore diverse language backgrounds, including adverse childhood communication experiences and later sign language age of exposure. Partnerships with DDBHH people with adequate research training will foster a more diverse and representative understanding of the role of emotion in language. By considering a more holistic role of emotion and cognition in language, we stand to improve social and educational practices in language development and encourage all learners to thrive.
